# Molecular Evidence of Dengue Virus Serotype 2 in Travelers Returning to Israel from the Sinai Peninsula

**DOI:** 10.3201/eid3111.250991

**Published:** 2025-11

**Authors:** Neta S. Zuckerman, Guy Choshen, Yaniv Lustig, Anna Shoykhet, Keren Friedman, Tatyana Kushnir, Ora Halutz, Hovav Azulay, Victoria Indenbaum, Eli Schwartz

**Affiliations:** Tel-Aviv University, Tel Aviv, Israel (N.S. Zuckerman, G. Choshen, Y. Lustig, O. Halutz, E. Schwartz); Sheba Medical Center, Ramat-Gan, Israel (N.S. Zuckerman, Y. Lustig, K. Friedman, T. Kushnir, V. Indenbaum, E. Schwartz); Tel-Aviv University, Tel Aviv, Israel (G. Choshen, Y. Lustig, O. Halutz, E. Schwartz); Meir Medical Center, Kfar-Saba, Israel (G. Choshen, A. Shoykhet); Infectious Disease Institute, Soroka University Medical Center, Beer Sheba, Israel (H. Azulay)

**Keywords:** dengue, dengue virus, viruses, vector-borne infections, dengue virus serotype type 2, DENV-2, next-generation sequencing, phylogenetic analysis, Israel, Sharm-El-Sheikh, Red Sea, Sinai Peninsula, Egypt

## Abstract

We report 4 dengue cases in travelers returning to Israel from Sharm-El-Sheikh, Egypt, all confirmed as dengue virus type 2 infections. Phylogenetic analysis showed clustering with strains from Pakistan. Our findings provide molecular evidence of dengue circulation in the Sinai desert, highlighting the need for increased awareness among travelers and health authorities.

Dengue virus (DENV) is the most widespread arbovirus globally; its incidence has increased tenfold in the past 2 decades, largely driven by climate change and globalization (*1*). Although transmission is well documented in Southeast Asia and the Americas, autochthonous emergence is increasingly reported in nonendemic regions, including Europe.

We report 4 confirmed dengue fever cases in travelers returning to Israel after visiting Sharm El-Sheikh, a desert resort city in South Sinai, Egypt, during April–June 2024. Sharm El-Sheikh has not previously been recognized as an area of dengue transmission, the arid environment of the Sinai Peninsula is considered unfavorable for the DENV primary vectors, *Aedes* mosquitoes.

The cases (Table) were unrelated; travel dates were nonoverlapping and accommodations varied and were located 3–25 km apart. Patients had typical dengue symptoms such as fever, headache, myalgia, and rash. All were hospitalized, received supportive care, and recovered. One patient exhibited meningeal irritation; cerebrospinal fluid testing results were unremarkable, although DENV serotype 2 (DENV-2) RNA was detected by quantitative real-time PCR (cycle threshold 32.5). All samples were collected within 1 week of symptom onset. Serum testing confirmed DENV-2 by multiplex quantitative real-time PCR (*2*); additional nonstructural protein 1 antigen and IgM/IgG positivity was detected in some cases.

**Table:** Epidemiology and test results of patients in study of molecular evidence of DENV-2 emergence from travelers returning to Israel from the Sinai Peninsula*

Characteristic	Patient 1	Patient 2	Patient 3	Patient 4
Patient age, y/sex	33/M	40/F	45/M	19/M
DENV laboratory diagnostic analysis				
Sample collection date	2024 Apr 22	2024 May 7	2024 Jun 5	2024 Jun 10
Serum quantitative real-time PCR	DENV-2 (Ct 28)	DENV-2 (Ct 26.5)	DENV-2 (Ct 27)	DENV-2 (Ct 34)
Serum EIA IgM	Negative	Negative	Positive	Positive
Serum EIA IgG	Negative	Negative	Positive	Negative
DENV nonstructural protein 1 antigen	Positive	Positive	Negative	Positive

*Ct, cycle threshold; DENV, dengue virus; DENV-2, DENV serotype 2; EIA, enzyme immunoassay.

To explore the geographic origin of the DENV-2 cases, we performed DENV whole-genome sequencing. We captured DENV-2 using specific whole-genome primers (https://grubaughlab.com/open-science/amplicon-sequencing); we prepared sequencing libraries using Nextera-XT and ran them on the Illumina NovaSeq (https://www.illumina.com). We generated consensus sequences by mapping to the DENV-2 reference genome (GenBank accession no. NC_001474.2) and deposited resulting sequences into GenBank (Appendix Table). Use of the samples in this study was approved by the Sheba Medical Center Institutional Review Board (approval no. SMC-6190-19).

Samples yielded sufficient DENV-2 genome coverage, except in the case of patient no. 4 (possibly because of high cycle threshold [34]), which was excluded. Phylogenetic analysis with global DENV-2 sequences (n = 1,492) clustered the Israel sequences within the Cosmopolitan genotype. All 3 sequences formed a distinct cluster, sharing a common ancestor and differing by 32 mutations from the nearest global strain. The closest related sequences were from Pakistan. The only publicly available sequence geographically close to Sinai, from the United Arab Emirates in 2023, clustered separately within another Cosmopolitan lineage with strains from China, India, and Bangladesh (Figure, panel A).

**Figure:**
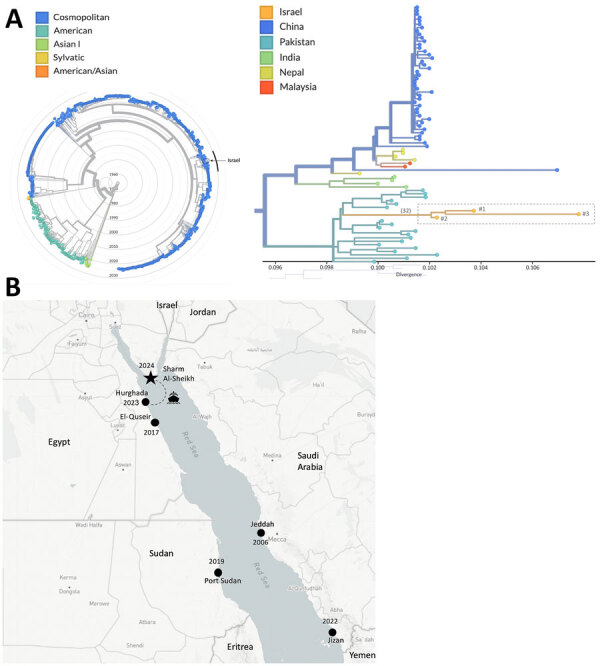
Epidemiology, phylogeny, and geography of dengue virus (DENV) serotype 2 (DENV-2) circulation in the Red Sea region in study of molecular evidence of DENV-2 emergence from travelers returning to Israel from the Sinai Peninsula. A) Phylogenetic analysis of DENV-2 whole-genome sequences. Phylogenetic tree was constructed using the samples from Israel sequenced in this study (n = 3) alongside global DENV-2 sequences from the past decade (n = 1,492) available in the DENV-2 Nextstrain build (https://nextstrain.org/dengue/denv2/genome). The radial tree illustrates the global DENV-2 genotypes, highlighting the Israel sequences (n = 3, circled) within the broader dataset. Nodes are color-coded by DENV genotype. The cluster marked by a black arc is magnified in the right portion. The rectangular tree focuses on the cluster containing the Israeli sequences (n = 3), showing the genetic divergence within the group. Nodes are color-coded by the country of origin, and the number of mutations connecting the Israeli cluster to the nearest ancestor is indicated in parentheses. B) Dengue fever outbreak locations, including the current outbreak in Sharm El-Sheikh (star) and outbreaks reported in the region in recent years (black circles labeled by year): Jeddah 2006 (*3*), El-Quseir 2017 (*4*), Port Sudan 2019 (*5*), Jizan 2022 (*6*), and Hurghada 2023 (*7*).

Our findings describe 4 confirmed DENV-2 infections in travelers from Sharm El-Sheikh, Egypt, a city in the arid Sinai Peninsula, previously considered unsuitable for *Aedes* mosquitoes and without previous dengue reports. Genomic analysis showed clustering of cases, likely from a single outbreak, most closely related to strains from Pakistan. Aside from 1 United Arab Emirates 2023 sequence clustering separately, no recent data from Sinai are available, underscoring a major surveillance gap. Those results align with reports of DENV-2 spread along the Red Sea and recent cases in Florence, Italy (*8*).

During the past 2 decades, *Ae. aegypti* mosquito populations have expanded in Egypt, especially along the Red Sea coast (Figure, panel B), correlating with dengue outbreaks. However, no entomologic data exist for Sinai. The arid climate challenges mosquito survival, but clustering of cases in 1 resort area suggests local adaptation, possibly supported by urban microhabitats (*9*). Maritime and air travel might drive repeated introductions of *Ae. aegypti* mosquitos and DENV into the Red Sea region. However, the pattern of DENV-2 outbreaks in Red Sea port cities support maritime transport as a key driver of spread (*6*,*7*,*10*). The daily ferries from Hurghada, where dengue recently emerged, to Sharm El-Sheikh might be especially relevant (Figure, panel B). Genetic data from a 2019 Jizan outbreak and strains from Saudi Arabia (1992–2014) further suggest multiple introductions linked to an imported DENV-2 variant genetically similar to strains from Malaysia, Singapore, Korea, and China (*6*). Additional analyses from the DENV-2 strains isolated in Saudi Arabia during 1992–2014 reveal strong clustering with viruses from countries that contribute the largest numbers of Hajj and Umrah pilgrims: Indonesia, Pakistan, and India (*10*). Indeed, phylogenetic analysis shows that our dengue sequences are closest to recent strains from Pakistan. However, the scarcity of sequences from Egypt and neighboring regions limits inference on viral origin, circulation, and distribution, and observed variability suggests undersampling and additional undetected cases.

This report of 4 cases over 3 months in different localities of Sharm El-Sheikh suggests sustained DENV-2 transmission and emphasizes the importance of enhanced vector surveillance and control, providing an alert to public health authorities. The genetic data presented might help address gaps in regional DENV sequence reporting and contribute to understanding its molecular epidemiology and origins.

Appendix Additional information about molecular evidence of dengue virus serotype 2 in travelers returning to Israel from the Sinai Peninsula.
